# Supplementary material for: Comparing In Vitro Virucidal Efficacy of Commercially Available Mouthwashes Against Native High-Risk Human Papillomavirus Types 16 and 18
Source: Microorganisms. 2025 Mar 25;13(4):734. doi: 10.3390/microorganisms13040734 (PMC12029689; doi:10.3390/microorganisms13040734)
Supplement: Supplementary file 1 [file microorganisms-13-00734-s001.zip › microorganisms-3486743-supplementary.pdf]

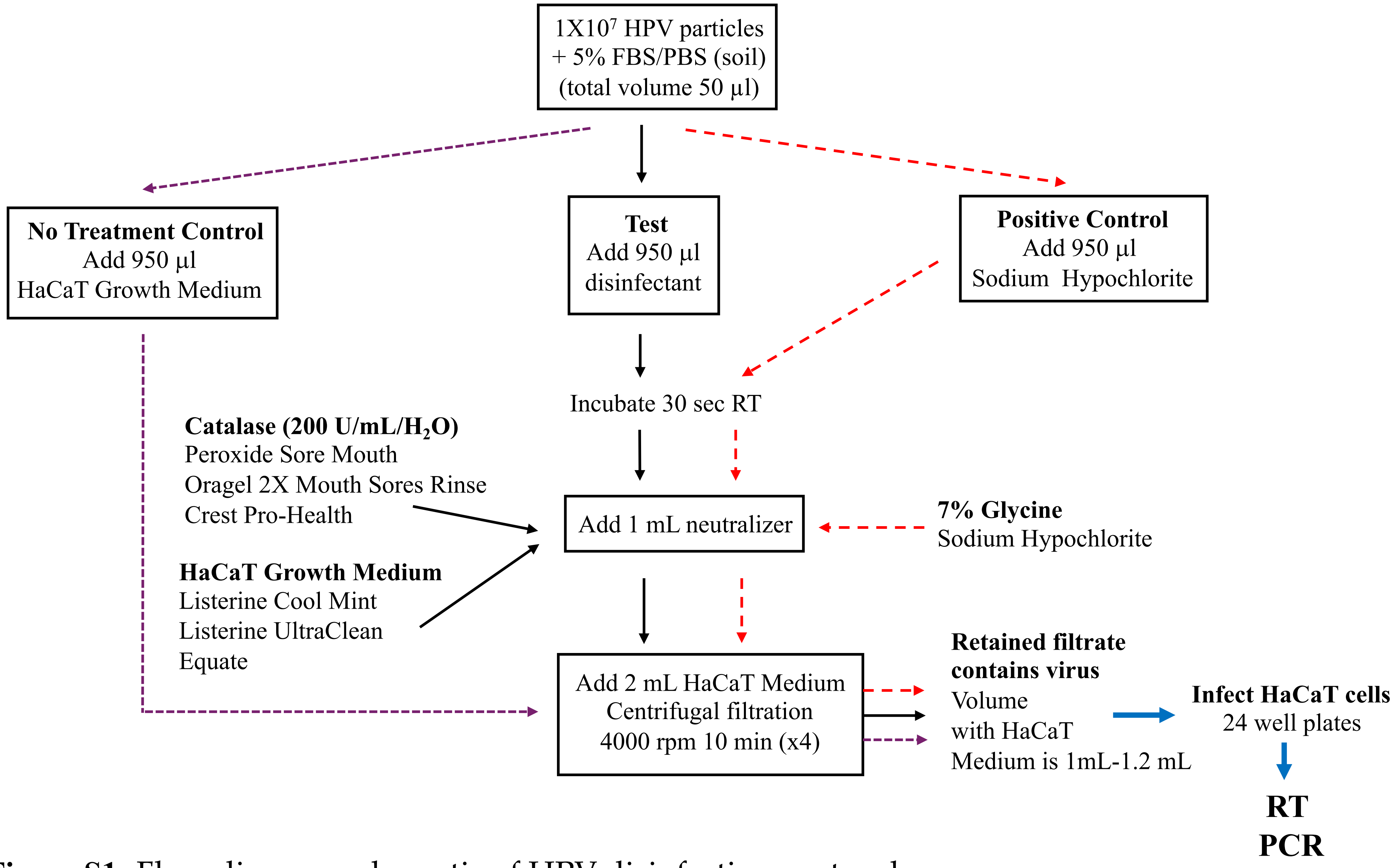

Figure S1: Flow diagram schematic of HPV disinfection protocol

Table S1
Primer and Probe Sequences

**HPV16 E2 5’**	5’ -CCA TAT AGA CTA TTG GAA ACA CAT GCG CC- 3’
**HPV16 E2 3’**	5’ -CGT TAG TTG CAG TTC AAT TGC TTG TAA TGC- 3’
**HPV16 E1^E4 5’**	5’ -GCT GAT CCT GCA AGC AAC GAA GTA TC- 3’
**HPV16 E1^E4 3’**	5’ -TTC TTC GGT GCC CAA GGC- 3’
**HPV18 E2 5’**	5’ -TCC GCT ACT CAG CTT GTT AAA CAG -3’
**HPV18 E2 3’**	5’ -CCC ACG GAC ACG GTG C -3’
**HPV18 E1^E4 5’**	5’ - GC TGA TC AGA AC CAG TGA -3'
**HPV18 E1^E4 3’**	5’ -CTG GC GTA GT CT TGC GT G-3'
**TBP 5’**	5’ -CAC GGC ACT GAT TTT CAG TTC T- 3’
**TBP 3’**	5’ -TTC TTG CTG CCA GTC TGG ACT- 3’
**Probe HPV16 E1^E4**	5’ -/56-FAM/CCC GCC GCG ACC CAT ACC AAA GCC/3BHQ-1/- 3’
**Probe HPV18 E1^E4**	5’ -/56-FAM/CCT CAC CGT AT CA GCA CG TGT CG TG/3BHQ_1/ -3'
**Probe TBP**	5’ -/5HEX/TGT GCA CAG GAG CCA AGA GTG AAG A/3BHQ-1/- 3’
